# The Effect of Topical Ketoconazole and Topical Miconazole Nitrate in Modulating the Skin Microbiome and Mycobiome of Patients With Tinea Pedis

**DOI:** 10.1111/myc.70116

**Published:** 2025-09-18

**Authors:** Yen Tan, Yakun Shao, Tingting Li, Xunyi Hu, Xiaowen Wang, Zhe Wan, Fuyou Yin, Ruoyu Li, Ruojun Wang

**Affiliations:** ^1^ Department of Dermatology Peking University First Hospital Beijing China; ^2^ National Clinical Research Center for Dermatologic and Immunologic Diseases Beijing China; ^3^ Research Center for Medical Mycology Peking University Beijing China; ^4^ Peking University Health Science Center Beijing China

**Keywords:** skin microbiome, skin mycobiome, topical antifungal agents

## Abstract

**Background:**

Tinea pedis is a type of dermatophytosis that affects the superficial layers of the skin on feet. Limited data are available on the skin microbiome composition in affected patients and its changes following topical antifungal therapy.

**Objectives:**

To evaluate the clinical and microbiological effects of topical ketoconazole 2% cream (KTZ) and miconazole nitrate 2% cream (MCZ) using standardised clinical scoring and amplicon sequencing.

**Methods:**

A total of 42 patients with tinea pedis and 28 healthy controls were enrolled. Skin swabs were collected from lesional sites (interdigital or heel) at baseline, after 4 weeks of treatment, and 2 weeks post‐treatment. DNA was extracted from the samples, and the bacterial 16S rRNA (V3–V4 region) and fungal ITS1–5F regions were sequenced to analyse microbial community composition.

**Results:**

Both KTZ and MCZ led to comparable clinical improvement. However, the KTZ group showed faster symptom resolution and a higher sustained improvement rate during follow‐up. Treatment with either antifungal effectively reduced the abundance of pathogenic *Trichophyton* species to levels similar to those in healthy controls, thereby contributing to partial recovery of the overall fungal community structure. In parallel, the bacterial profile became more dispersed, with notable shifts observed in bacterial genera such as *Staphylococcus* and *Corynebacterium* following treatment.

**Conclusion:**

Topical antifungal therapy with KTZ or MCZ effectively improved the symptoms of tinea pedis, diminished the pathogenic fungal load and altered both fungal and bacterial community compositions. However, only partial restoration of the mycobiome was achieved, and the bacterial profile, especially in the interdigital region, showed a lack of bacterial normalisation. These findings highlight the need for further studies to assess long‐term outcomes and to explore microbiome‐targeted strategies addressing both bacterial and fungal components.

## 1 Introduction

Tinea pedis, often known as ‘athlete's foot’, is a common superficial mycosis of the feet, primarily caused by dermatophytes. The global prevalence of tinea pedis is estimated to be about 3% of the world population, with a lifetime prevalence reaching up to 70% [1]. Clinically, it is characterised by erythema, scaling, fissuring, pruritus, and maceration. The infection typically involves the interdigital spaces or the soles of the feet [2, 3, 4, 5]. While the predominant dermatophyte species may vary by geographic region, the most commonly reported causative agents belong to the *Trichophyton* (*T*.) genera [6, 7, 8, 9].

Increasing evidence suggests that bacterial involvement contributes to the progression and severity of tinea pedis, potentially affecting dermatophyte detection, antifungal susceptibility, and the recurrence of disease course [10, 11, 12]. An increased prevalence of *Staphylococcus* (*S*.) *aureus, Streptococcus, Corynebacterium minutissimum*, and Gram‐negative bacteria such as *Pseudomonas* has been reported in the interdigital space of patients with tinea pedis [10, 12, 13, 14, 15]. Microbiome differences have also been observed between interdigital and plantar foot specimens [13].

Topical antifungal therapy is the first‐line treatment for localised or superficial tinea pedis [1, 16, 17, 18]. Most uncomplicated cases respond well to topical broad‐spectrum azole agents. Azole antifungals exert their effect by inhibiting cytochrome P‐450‐dependent enzyme lanosterol demethylase (P‐450_DM_), which is essential for the biosynthesis of ergosterol, a key component of fungal plasma membranes. Imidazoles, a subclass of azoles characterised by two nitrogens in the azole ring, include agents such as ketoconazole and miconazole. These drugs are widely used and have demonstrated efficacy in treating superficial mycoses, including tinea pedis [16, 19, 20]. However, studies investigating microbiome changes following antifungal treatment are scarce.

To elucidate the cutaneous microbial profile in tinea pedis and its regional variations, as well as to assess alterations in both bacterial and fungal communities following antifungal therapy, we herein conducted a prospective cohort study. By characterising the skin microbiota of patients with tinea pedis before and after topical treatment with ketoconazole or miconazole, and by separately analysing samples from the interdigital and heel regions, our study goal was to investigate the modulatory effects of these antifungal agents on the skin microbiome and to identify skin region‐specific microbial differences.

## 2 Methods

### 2.1 Patient Recruitment and Randomisation

Patients with tinea pedis and the healthy controls were recruited from the dermatology clinic in Peking University First Hospital between May 2023 and May 2024. The study protocol has been reviewed and received ethical approval from the Institutional Review Board of Peking University First Hospital, Beijing, China (Number: 2023170). Tinea pedis was diagnosed based on typical clinical manifestations and a positive microscopic examination. Exclusion criteria included a history of concomitant dermatoses on the foot, malignancies, autoimmune diseases, or severe diabetes, and any use of topical or systemic antibiotics, antifungal agents, and immunosuppressants within 12 weeks prior to the study.

Patients were randomly assigned (1:1) to receive topical ketoconazole 2% cream (NIZORAL Cream, Johnson & Johnson Innovative Medicine, Belgium) in comparison to topical miconazole nitrate 2% cream (DAKTARIN Cream, Johnson & Johnson Innovative Medicine) with a computer‐generated randomisation schedule using Microsoft Excel.

### 2.2 Disease Severity Evaluation

Disease severity was assessed before and after treatment using an Athlete's foot severity score (AFSS) system [21]. It evaluates five key clinical signs: erythema, papulation, blister or vesicle, maceration and scaling. Each of these signs is scored on a scale from 0 to 3, with 0 indicating no presence of the sign and 3 indicating the most severe presence. We evaluated treatment efficacy using B%, the improvement rate calculated as: B% = (severity score before treatment A1 − severity score after treatment A2)/A1 × 100%. A B% ranging from 91% to 100% is considered cured, 51% to 90% is significantly improved, 11% to 50% is improved and 0% to 10% is not improved.

In addition, itching score was evaluated by patient self‐assessment using a Peak Pruritus Numerical Rating Scale (PP‐NRS). Time to cure was defined as the point at which the patient's AFSS score reached 0.

### 2.3 Sample Collection and Processing

Skin swabs of lesional sites (interdigital or heel site) were obtained using a sterile cotton swab premoistened in phosphate‐buffered saline (PBS). Blank controls were prepared similarly using sterile swabs premoistened with PBS but not applied to any sampling site. The head of each swab was cut from the handle and placed into a tube containing 1.5 mL of PBS. The swab samples were stored at −20°C before DNA extraction.

DNA was extracted from skin samples using the Biospin Genomic DNA extraction kit (BioFlux, China). The quality and concentration of the extracted DNA were evaluated using a Nanodrop 2000 spectrophotometer (ThermoFisher Scientific, Waltham, MA, USA) as previously described [22]. The DNA was stored at −20°C prior to PCR analysis.

### 2.4 Amplicon Sequencing and Bioinformatic Analysis

Fungal DNA was amplified by targeting the internal transcribed spacer (ITS1) region through specific primers (forward ITS5‐1737F: 5′‐GGAAGTAAAAGTCG TAACAAGG‐3′; reverse ITS2‐2043R: 5′‐GCTGCGTTCTTCATCGATGC‐3′). For bacterial profiling, the V3–V4 region of the 16S rRNA gene was amplified using primers (forward 341F: 5′‐TCGTCGGCAGCGTCAGATGTGTATAAGAGACAGCCTACGGGNGGCWGCAG‐3′; reverse 805R: 5′‐GTCTCGTGGGCTCGGAGATGTGTATAAGAGACAGGACTACHVGGGTATCTAATCC‐3′). PCR products were purified, and sequencing libraries were prepared, and quality assessments were performed as previously described [14, 23]. Paired‐end sequencing was performed on an Illumina HiSeq 2500 platform by an external company (Novogene Bioinformatics Technology Co. Ltd., Beijing, China). Sequence data from this study have been deposited in NCBI under accession number PRJNA1285305.

Quality filtering and clustering were performed using QIIME2 using default parameters for each step [24]. Representative sequences were screened and utilised for the annotation of taxonomic information using the Unite database (https://unite.ut.ee/) for ITS rDNA data and the SILVA (http://www.arb‐silva.de/) SSU rRNA Database for V3V4 region of the 16S rRNA data with QIIME2.

The Shannon diversity was calculated to evaluate the richness and evenness of microbial communities using the vegan package in R software (Version 3.6.1). Non‐metric Multidimensional Scaling (NMDS) was plotted to visualise the clustering patterns among samples using R.

### 2.5 Statistical Analysis

All data analyses were performed using R software (Version 3.6.1). For all analyses, comparisons were made between data at baseline and controls, baseline and visit 2 (after 4 weeks of treatment), and baseline and visit 3 (2 weeks post‐treatment). Continuous data with a normal distribution, including age, disease severity indexes, and itch index, were assessed using Student's *t*‐test for two‐group comparisons and Chi‐squared test for multigroup comparisons. For microbiome data without a normal distribution, differences were assessed using Wilcoxon rank sum test for two‐group comparisons and Kruskal‐Wallis test for multigroup comparisons.

## 3 Results

### 3.1 Patient Characteristics

Overall, 42 patients with tinea pedis and 28 non‐tinea pedis controls were enrolled in this study. No significant differences in age, sex ratio, and baseline disease severity were observed between groups (Table 1). No side effects were detected in this study.

**TABLE 1:** Demographics of participants.

Characteristics	KTZ (*n* = 20)	MCZ (*n* = 22)	Control (*n* = 28)	*p*
Age, mean (SD), years	40.11 (12.22)	47.78 (13.06)	41.75 (15.55)	NS
Male	11 (19)	17 (21)	14 (28)	NS
Sampling site				
Interdigital space	11	12	21	NS
Heel	9	10	7	
Disease severity, mean (SD)	5.85 (2.23)	5.27 (2.49)	NA	NS
pp‐NRS, mean (SD)	4.05 (3.49)	3.41 (2.86)	NA	NS

Abbreviations: KTZ, ketoconazole; MCZ, miconazole nitrate; NA, not available; NS, not significant; pp‐NRS, peak pruritus numerical rating scale; SD, standard deviation.

### 3.2 Topical Ketoconazole Outperforms Topical Miconazole Nitrate in the Course of Treatment for Tinea Pedis

**FIGURE 1:**
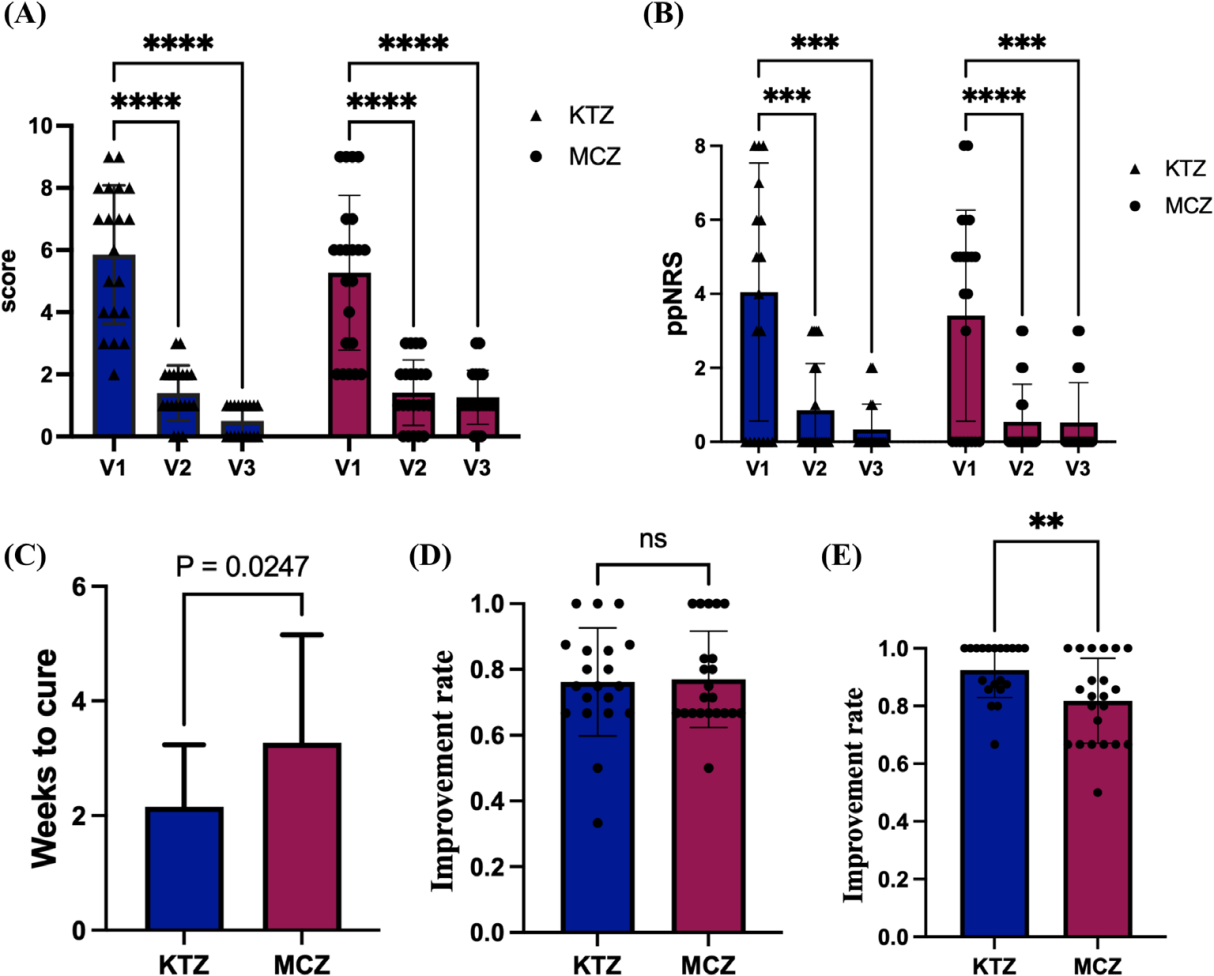
The disease severity (A, evaluated by AFSS) and itching score (B) and at baseline (V1), 4 weeks after treatment (V2) and 2 weeks posttreatment (V3) in two groups. (C) Patient‐reported time to cure in the KTZ‐ and MCZ‐treated groups. Improvement rate after 4 weeks of treatment (D) and at 2 weeks posttreatment (E) in the KTZ‐ and MCZ‐treated groups.

Disease severity assessed using the AFSS showed that both KTZ and MCZ significantly decreased the severity score after 4 weeks of treatment, and these improvements were maintained for 2 weeks post‐treatment (Figure 1A). Analysis of the itch scores in patients with tinea pedis revealed a decrease in pp‐NRS levels following topical antifungal treatment (Figure 1B).

By comparing the AFSS scores, we further analysed the time to cure in different groups and revealed that the KTZ group had a significantly shorter time to symptom remission compared to the MCZ group (*p* < 0.05) (Figure 1C). Analysis of the improvement rate at different time points revealed that the KTZ group (76.21% ± 16.39%) had a similar improvement rate as the MCZ group (77.02% ± 9.64%) after 4 weeks of topical treatment. However, 2 weeks after drug withdrawal, the improvement rate of the KTZ group (92.54% ± 9.64%) was significantly higher than that of the MCZ group (81.86% ± 14.74%) (*p* < 0.01) (Figure 1D,E).

### 3.3 Distinct Skin Microbiome Between Interdigital Space and Heel

**FIGURE 2:**
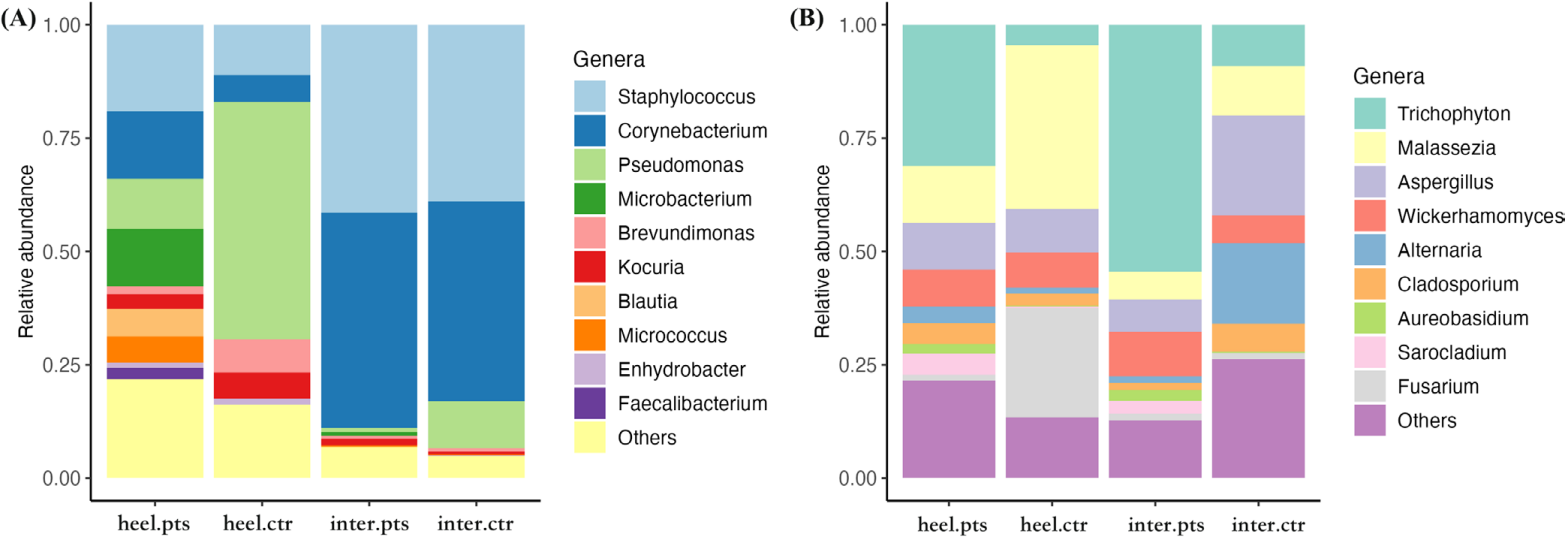
The skin microbiome (A) and mycobiome (B) baseline data of patients with tinea pedis and controls. heel.ctr, heel of controls; heel.pts, heel of tinea pedis patients; inter.ctr, interdigital space of controls; inter.pts., interdigital space of tinea pedis patients.

Considering various factors, such as humidity, temperature and physical maceration, that may influence the microbiome of different foot regions [25, 26, 27, 28], we first conducted an analysis comparing the baseline skin microbiome and mycobiome structures across different foot areas (interdigital space vs. heel). The results revealed a similar fungal community structure across foot regions in healthy controls, with only a significant difference noted in *Fusarium* (*p* < 0.01, Table 2, Figure 2). The fungal composition changed drastically in patients with tinea pedis, and significant differences were observed in *Malassezia*, *Alternaria* and *Cladosporium* between interdigital and heel areas (*p* < 0.05, Table 2). A slightly higher abundance of *Trichophyton* was also observed in tinea pedis patients in the interdigital space compared to the heel, although this difference was not statistically significant (Table 2).

**TABLE 2:** Characterisation of the skin microbiome in different regions in healthy controls and tinea pedis patients at baseline.

Taxonomy	heel.ctr	inter.ctr	*p* ^a^	heel.pts.	inter.pts	*p* ^b^
Fungal genus						
*Candida*	0.1221	0.1810	NS	0.1686	0.1537	NS
*Trichophyton*	0.0393	0.0745	NS	0.2594	0.4607	NS
*Malassezia*	0.3176	0.0889	NS	0.1039	0.0519	< 0.05
*Aspergillus*	0.0842	0.1807	NS	0.0859	0.0602	NS
*Wickerhamomyces*	0.0679	0.0504	NS	0.0675	0.0835	NS
*Alternaria*	0.0112	0.1453	NS	0.0307	0.0122	< 0.05
*Cladosporium*	0.0239	0.0510	NS	0.0378	0.0130	< 0.05
*Aureobasidium*	0.0019	0.0018	NS	0.0178	0.0203	NS
*Sarocladium*	0.0027	0.0005	NS	0.0387	0.0237	NS
*Fusarium*	0.2120	0.0109	< 0.01	0.0108	0.0123	NS
Others	0.1172	0.2151	NS	0.1790	0.1084	NS
Bacterial genus						
*Staphylococcus*	0.1105	0.3894	< 0.01	0.1913	0.4154	< 0.01
*Corynebacterium*	0.0598	0.4407	< 0.001	0.1485	0.4732	< 0.001
*Pseudomonas*	0.5230	0.1038	< 0.001	0.1103	0.0094	< 0.01
*Microbacterium*	0.0000	0.0000	NS	0.1271	0.0089	< 0.01
*Brevundimonas*	0.0736	0.0074	< 0.01	0.0177	0.0060	NS
*Kocuria*	0.0576	0.0064	NS	0.0320	0.0146	NS
*Blautia*	0.0000	0.0001	< 0.05	0.0609	0.0000	NS
*Micrococcus*	0.0000	0.0017	< 0.01	0.0574	0.0029	< 0.05
*Enhydrobacter*	0.0131	0.0023	NS	0.0109	0.0000	< 0.01
*Faecalibacterium*	0.0000	0.0003	NS	0.0249	0.0000	NS
Others	0.1625	0.0478	< 0.01	0.2189	0.0696	< 0.05

Abbreviations: Ctr, control; inter, interdigital space; NS, not significant; Pts, patients.

^a^ Comparison of the heel and interdigital skin microbiome in healthy controls.

^b^ Comparison of the heel and interdigital skin microbiome in tinea pedis patients at baseline.

In contrast, analysis of the skin bacterial microbiome from different sites revealed more distinct community structures, primarily characterised by a significantly higher abundance of *Staphylococcus* (*p* < 0.01, Wilcoxon test) and *Corynebacterium* (*p* < 0.001, Wilcoxon test) in the interdigital space compared to the heel, in both healthy controls and patients with tinea pedis (Table 2, Figure 2).

These discrepancies in the baseline data highlight the necessity to analyse the therapeutic effects on the microbiome separately for different foot areas in subsequent analyses.

### 3.4 Topical Antifungal Agents Diminished the Abundance of Pathogenic Fungi

**FIGURE 3:**
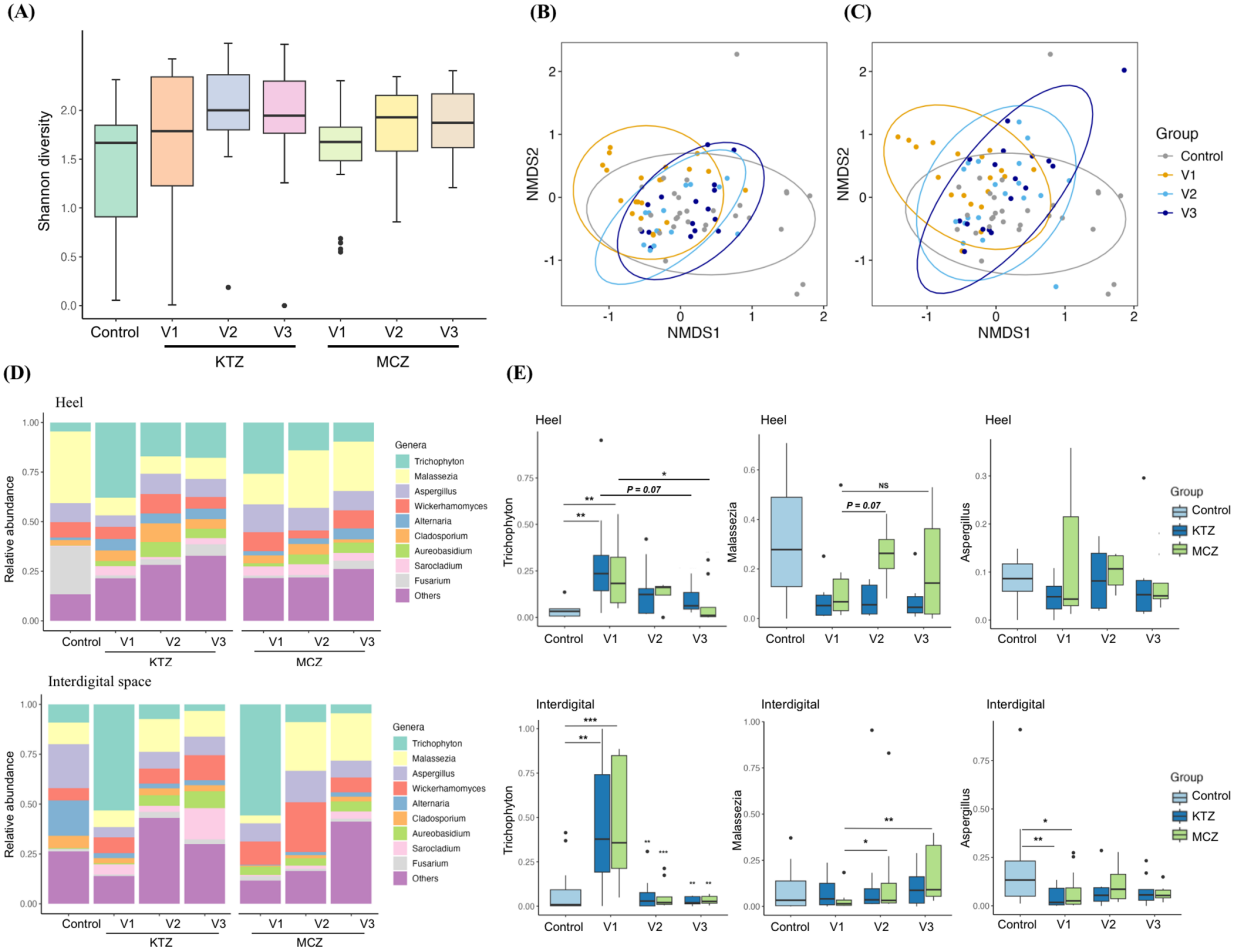
(A) Shannon diversity of the skin mycobiome for patients with tinea pedis at baseline (V1), 4 weeks after treatment (V2) and 2 weeks posttreatment (V3) and controls. (B, C) NMDS plots of the fungal community in topical KTZ treatment (B) and topical MCZ treatment (C) among baseline, after treatment, 2 weeks posttreatment and controls. (D) Skin mycobiome structures of patients with tinea pedis at different time points and controls for heel and interdigital spaces. (E) The relative abundances of *Trichophyton*, *Malassezia* and *Aspergillus* in tinea pedis patients at different time points and controls for heel and interdigital spaces.

Next, we analysed the skin fungal community structure between patients and controls in different foot regions. As expected, a significantly higher relative abundance of *Trichophyton* was observed in patients with tinea pedis at both interdigital spaces and heels at baseline (Figure 3E), which predominantly comprised *T. rubrum* at the species level (Figure S3A). In the interdigital region, both KTZ and MCZ treatment groups showed significantly decreased abundance of *Trichophyton* after four weeks of topical treatment and two weeks post‐treatment, whereas the heel region exhibited a similar decreasing trend that did not reach statistical significance (Figure 3E). The levels of *T. rubrum* were also significantly reduced following treatment with either topical KTZ or MCZ and remained low two weeks after treatment cessation (*p* < 0.01, Figure S3B).

In addition, an increased abundance of *Malassezia* was observed in all patients with tinea pedis treated with MCZ, both after the treatment and during post‐treatment follow‐up visits, with the rise more evident in the interdigital region, a pattern not observed in the KTZ group. A decreased abundance of *Aspergillus* was also observed in the interdigital region of patients with tinea pedis at baseline compared to healthy controls, but this did not change significantly following treatment (Figure 3E).

Analysis of the fungal microbiome alpha diversity revealed no significant differences in Shannon diversity among tinea pedis patients at different time points and controls (Figure 3A), as well as in region‐specific analyses (Figure S1A,B). NMDS analysis of the fungal communities at the genus level revealed that both KTZ‐ and MCZ‐treated patients showed partial overlap and incomplete separation from controls after treatment, indicating a fungal community composition similar to that of healthy individuals after treatment with topical antifungal agents (Figure 3B,C). Furthermore, region‐specific NMDS analysis revealed that the heel regions of patients exhibited greater overlap with healthy controls after treatment compared to the interdigital regions (Figure S1C,D).

### 3.5 Topical Antifungal Agents Reshape the Skin Bacterial Microbiome

**FIGURE 4:**
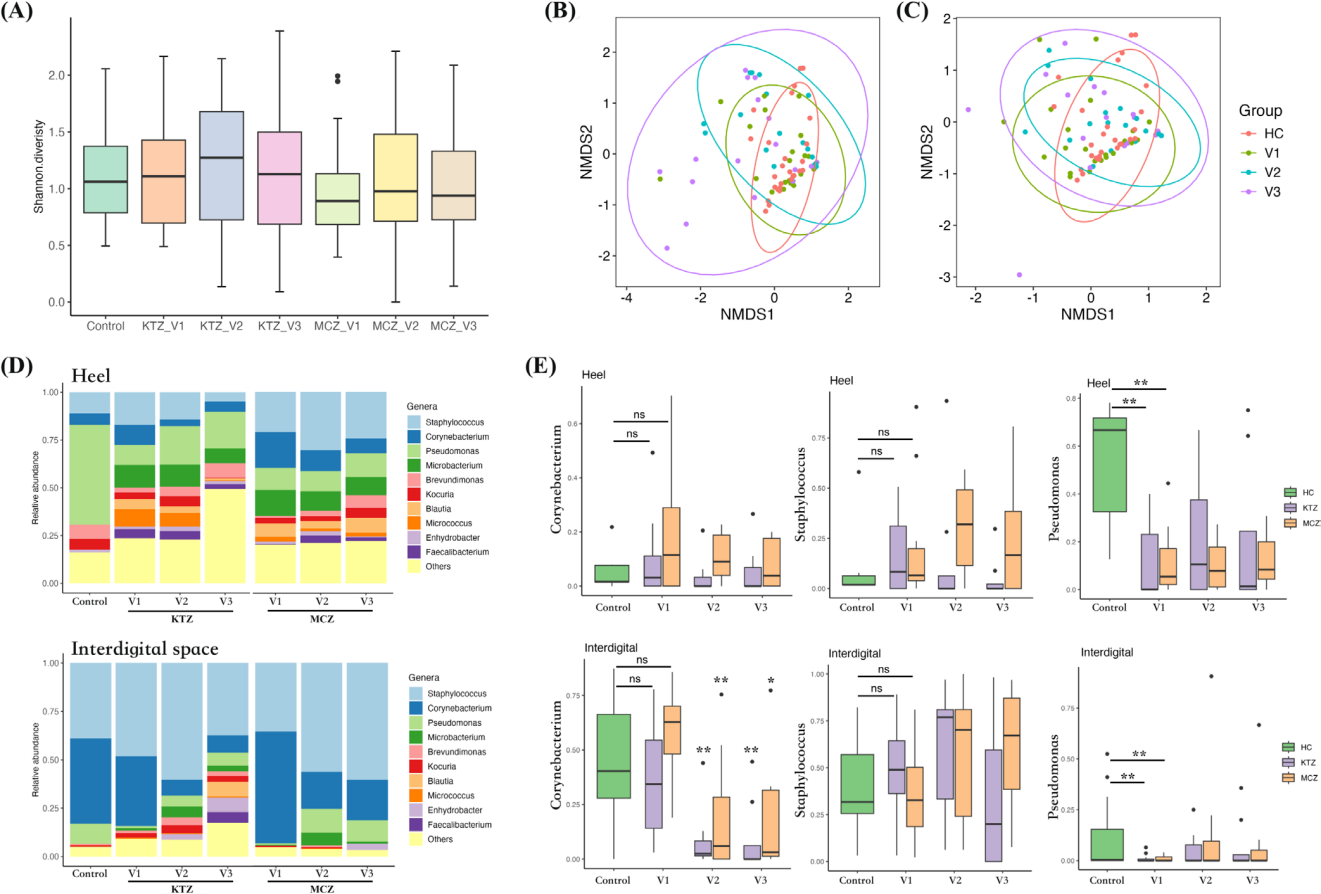
(A) Shannon diversity of the skin microbiome for patients with tinea pedis at baseline (V1), 4 weeks after treatment (V2) and 2 weeks post‐treatment (V3) and controls. (B, C) NMDS plots of the bacterial community in topical KTZ treatment (B) and topical MCZ treatment (C) among baseline, after treatment, 2 weeks post‐treatment and controls. (D) Skin bacterial structures of patients with tinea pedis at different time points and controls for heel and interdigital spaces. (E) The relative abundances of *Staphylococcus*, *Corynebacterium* and *Pseudomonas* in tinea pedis patients at different time points and controls for heel and interdigital spaces.

Given the distinct baseline bacterial communities across different foot areas, we also analysed the skin microbiome separately following topical antifungal treatment. Our results revealed that both topical KTZ and MCZ significantly reduced the relative abundance of *Corynebacterium* at the interdigital spaces after treatment and at the 2‐week follow‐up, despite the absence of significant baseline differences between tinea pedis patients and healthy controls (*p* > 0.05). Interestingly, although *Staphylococcus* abundance was higher in the interdigital and heel regions of patients compared to controls (not statistically significant), it exhibited opposing trends following treatment: KTZ treatment was associated with a non‐significant decrease, whereas MCZ treatment appeared to further increase its abundance. In addition, *Pseudomonas*, a Gram‐negative bacterium, showed significantly lower abundance in patients in both regions at baseline, with no significant change following topical treatment (Figure 4E).

Overall, there was no significant difference in the bacterial Shannon diversity between patients with tinea pedis and controls, nor among patients at baseline, after treatment, and two weeks post treatment (Figure 4A). Similar results were observed in separate analyses of the heel and interdigital regions (Figure S2A,B). Although the overall NMDS analysis showed a more dispersed bacterial community structure after both treatments, the profiles did not shift closer to that of the controls (Figure 4B,C). Region‐specific analysis revealed that the interdigital area results were similar to the overall pattern, with a more dispersed bacterial community after treatment; however, in the heel region, NMDS plots for both KTZ and MCZ treatment showed partial overlap with incomplete separation between patients and controls (Figure S2C,D).

## 4 Discussion

The skin is inhabited by a complex and diverse microbiota [29]. Dermatophytes, the primary pathogens responsible for tinea pedis, damage the stratum corneum by secreting proteolytic enzymes. When the skin barrier is compromised, normally harmless bacteria residing in the toe web spaces can thrive and proliferate as moisture levels rise, transforming a purely fungal infection (dermatophytosis simplex) into a mixed fungal‐bacterial infection (dermatophytosis complex). The overgrowth of bacteria contributes to inflammation, maceration, and malodor, and produces fungicidal sulfur compounds that may drive dermatophytes into deeper levels of the stratum corneum. As bacterial levels decline, the dermatophytes migrate back towards the surface, reverting the infection from the complex to the simplex form [10, 30]. This dynamic highlights the polymicrobial nature of the foot microbiome and underscores the importance of understanding fungal–bacterial interactions in the management of tinea pedis [31, 32, 33].

In this prospective cohort study, we employed clinical assessment tools and patient‐reported outcomes to evaluate the efficacy of 4‐week topical treatments with KTZ and MCZ. We also performed amplicon sequencing to characterise the skin fungal and bacterial communities before and after treatment. Our results demonstrated that both antifungal agents effectively alleviated symptoms and reduced the abundance of the pathogenic fungi *Trichophyton*. To our knowledge, this is the first study to characterise the dynamic changes in the skin microbiome before and after topical imidazole treatment in patients with tinea pedis.

Clinically, both KTZ and MCZ significantly reduced the clinical severity of tinea pedis after 4 weeks of topical application, with improvements being sustained at the 2‐week follow‐up. These two treatments exhibited comparable and consistent improvement rates with previous clinical trials; however, more rapid clinical recovery and a significantly higher improvement rate were observed in the KTZ group at the post‐treatment visit [34, 35, 36, 37, 38, 39, 40, 41, 42]. This enhanced efficacy may be attributed to KTZ's preferential binding to keratinocytes and its prolonged activity in the stratum corneum despite similar percutaneous absorption profiles among imidazoles [34, 43].

*T. rubrum* and *T. interdigitale* are recognised as the two primary causative pathogens of tinea pedis; however, their prevalence varies geographically [44]. Our data showed significantly elevated baseline levels of *Trichophyton* in both foot regions, with *T. rubrum* identified as the predominant etiological agent, consistent with most microbiome studies conducted in China [44, 45]. Following four weeks of topical KTZ or MCZ treatment, *Trichophyton*, specifically *T. rubrum* , declined to levels comparable to controls and remained low for at least two weeks post‐treatment, supporting the efficacy of both agents in eliminating the pathogenic fungi and aligning with earlier studies that reported negative fungal cultures or microscopy results after treatment [34, 35, 36, 37, 38, 39, 40, 41, 42, 46, 47, 48, 49, 50]. Interestingly, we also observed an increase in *Malassezia* in both foot regions following MCZ treatment and at subsequent follow‐up, a pattern not seen in the KTZ group. This rise may be attributable to MCZ's limited inhibitory effect on *Malassezia* [51, 52] and the reduction of *Trichophyton*, which together likely contributed to ecological release and allowed *Malassezia* to expand. The consistently low levels of *Aspergillus* in the interdigital region may similarly reflect colonisation resistance exerted by *Trichophyton* [53]. By conducting separate analyses of fungal NMDS plots in different foot regions, we revealed that the heel areas of patients post‐treatment exhibited closer clustering with healthy controls, suggesting a relatively better recovery of the fungal community at this site after treatment compared to the interdigital region. This difference is likely due to the lower baseline abundance of *Trichophyton* in the heel prior to treatment, highlighting distinct fungal recovery patterns across anatomical sites.

Interdigital web space is a warm and moist environment that promotes the growth of *Corynebacterium* and *Staphylococcus*, whereas the plantar heel has a thicker epidermis and lower moisture levels, leading to colonisation mainly by *Firmicutes*, particularly *Staphylococcus* [25, 30, 54]. In comparison with previously reported data, the abundance of *Staphylococcus* was markedly lower in the healthy plantar region than in interdigital areas in healthy individuals [15, 55]. In line with these findings, our study identified *Staphylococcus and Corynebacterium* as the predominant bacterial genera in the interdigital area of the control group, with abundances much higher than those in the heel area. The regional differences in bacterial distribution across the foot in healthy individuals further highlight the necessity of separately analysing alterations in the microbiome structure following treatment in tinea pedis patients.

The bacterial‐dermatophyte interplay on the foot has been explored in previous studies [12, 44]. In a study analysing interdigital lesions from patients with clinically suspected superficial mycoses and/or erythrasma, *Corynebacterium* spp. was frequently isolated alongside *T. rubrum* , *T. mentagrophytes*, and *Trichosporon*, suggesting that *Corynebacterium* often coexists with dermatophytes [15, 56, 57]. Besides that, *Streptococcus* was significantly enriched in the patient cohort compared with both healthy and remission groups [14]. Furthermore, *S. aureus* was isolated in 28.2% of tinea pedis patients [58], and was the most common isolated pathogen aside from dermatophytes [13]. *Pseudomonas* was also found to be negatively correlated with *Trichophyton* [57]. These findings suggested that potential interactions were ongoing between the fungal and bacterial species in tinea pedis.

The antibacterial therapy in treating tinea pedis has garnered increasing attention, as this disease is recognised as a fungal‐bacterial dysbiosis, rather than merely a proliferation of pathogenic fungi [59, 60]. MCZ can disrupt bacterial membrane structure and permeability, while KTZ slows bacterial growth by altering fatty acid composition [61]. Both topical agents exhibit activity against Gram‐positive bacteria, with MCZ showing particularly high efficacy against *Corynebacterium* spp. (MIC as low as 0.39 μg/mL) [61, 62, 63]. In vivo, 2% topical KTZ has also been shown to exert antibacterial effects and anti‐inflammatory activity, possibly through inhibition of 5‐lipoxygenase [64, 65].

In our study, although there were no significant differences in the baseline levels of *Corynebacterium* and *Staphylococcus* between patients and controls in the interdigital area, both KTZ and MCZ treatments significantly reduced *Corynebacterium* abundance, with a declining trend in *Staphylococcus* observed in the KTZ group. These changes, particularly the reduction in *Corynebacterium*, are consistent with the known antibacterial properties of both drugs, though indirect effects driven by shifts in the fungal community cannot be excluded. Additionally, *Pseudomonas*, which showed lower baseline abundance in patients in both foot regions and remained suppressed after treatment, may have been competitively excluded by the dominance of *Corynebacterium* and *Staphylococcus* [66, 67]. While these bacterial changes were evident, NMDS analyses indicated that topical antifungal treatment did not result in a bacterial profile resembling that of healthy controls, especially in the interdigital region, suggesting that incomplete normalisation, potentially resulting from direct drug effects or adaptive bacterial responses, may contribute to the recurrence of tinea pedis.

To minimise potential bias, age and sex were matched between groups, consistent sampling methods and skin sites were used, and detailed clinical data were recorded for each patient. Study limitations include a relatively small sample size and short follow‐up duration. The limited post‐treatment follow‐up duration may have missed potential microbiome rebound, which could contribute to recurrence. External factors such as physical activity and footwear type, which may influence microbial composition, were not controlled in this study. Longer‐term studies with more controlled factors are needed to better understand the trajectory of microbial recovery and disease dynamics.

## 5 Conclusion

Topical treatment with KTZ or MCZ effectively alleviated symptoms of interdigital tinea pedis and significantly reduced the abundance of the pathogenic genus *Trichophyton*. Compared to MCZ, KTZ presented a more rapid onset of symptom relief and a higher sustained improvement rate after treatment cessation. These antifungal therapies not only successfully altered the fungal community by partially restoring it to a healthy state, but also influenced the bacterial components, particularly reducing *Corynebacterium* and *Staphylococcus*. Further studies with larger cohorts and longer follow‐up would help to better understand the long‐term effects of antifungal therapy on the skin microbiome and its role in disease prevention and recurrence.

## Author Contributions

**Yen Tan:** writing – review and editing, writing – original draft, investigation. **Yakun Shao:** investigation, resources. **Tingting Li:** methodology, resources, investigation. **Xunyi Hu:** investigation. **Xiaowen Wang:** investigation. **Zhe Wan:** investigation. **Fuyou Yin:** investigation. **Ruoyu Li:** conceptualization, funding acquisition, supervision. **Ruojun Wang:** conceptualization, data curation, formal analysis, methodology, writing – review and editing, supervision.

## Conflicts of Interest

The authors declare no conflicts of interest.

## Supporting information


**Appendix S1:** myc70116‐sup‐0001‐AppendixS1.docx.

## Data Availability

The data that support the findings of this study are openly available in NCBI at https://www.ncbi.nlm.nih.gov/bioproject/PRJNA1285305/, reference number PRJNA1285305.
