# Supplementary material for: The Effect of Topical Ketoconazole and Topical Miconazole Nitrate in Modulating the Skin Microbiome and Mycobiome of Patients With Tinea Pedis
Source: Mycoses. 2025 Sep 18;68(9):e70116. doi: 10.1111/myc.70116 (PMC12444619; doi:10.1111/myc.70116)
Supplement: Supplementary file 1 — Appendix S1: myc70116‐sup‐0001‐AppendixS1.docx. [file MYC-68-e70116-s001.docx]

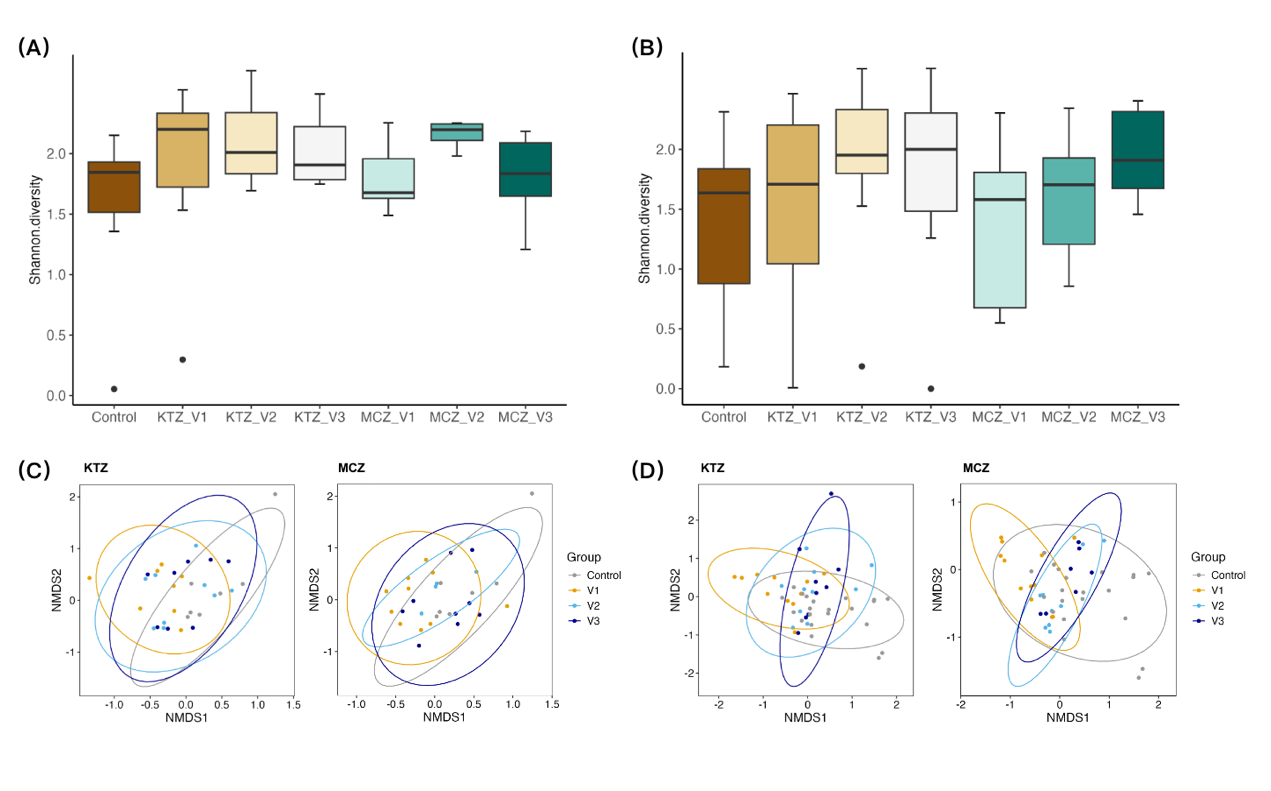
 Supplementary figure 1. (A) Shannon diversity of the heel skin fungal microbiome in controls and tinea pedis patient at baseline (V1), 4 weeks after treatment (V2) and 2 weeks posttreatment (V3). (B) Shannon diversity of the interdigital skin fungal microbiome in controls and tinea pedis patient at baseline (V1), 4 weeks after treatment (V2) and 2 weeks posttreatment (V3). (C) NMDS plots of the heel skin fungal community in topical KTZ and MCZ treatments across baseline, after treatment, 2 weeks post-treatment and controls. (D) NMDS plots of the interdigital skin fungal community in topical KTZ and MCZ treatments across baseline, after treatment, 2 weeks post-treatment and controls.


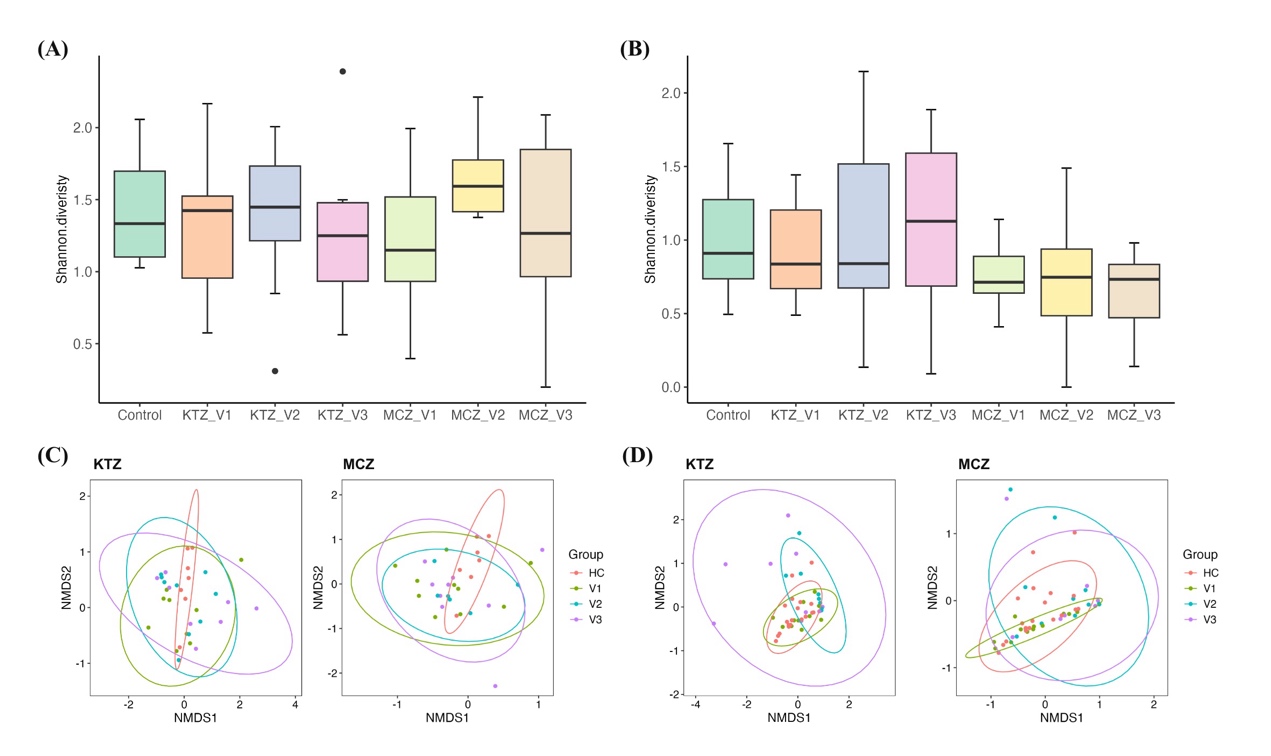


Supplementary figure 2. (A) Shannon diversity of the heel skin bacterial microbiome in controls and tinea pedis patient at baseline (V1), 4 weeks after treatment (V2) and 2 weeks posttreatment (V3). (B) Shannon diversity of the interdigital skin bacterial microbiome in controls and tinea pedis patient at baseline (V1), 4 weeks after treatment (V2) and 2 weeks posttreatment (V3). (C) NMDS plots of the heel skin bacterial community in topical KTZ and MCZ treatments across baseline, after treatment, 2 weeks post-treatment and controls. (D) NMDS plots of the interdigital skin bacterial community in topical KTZ and MCZ treatments across baseline, after treatment, 2 weeks post-treatment and controls.


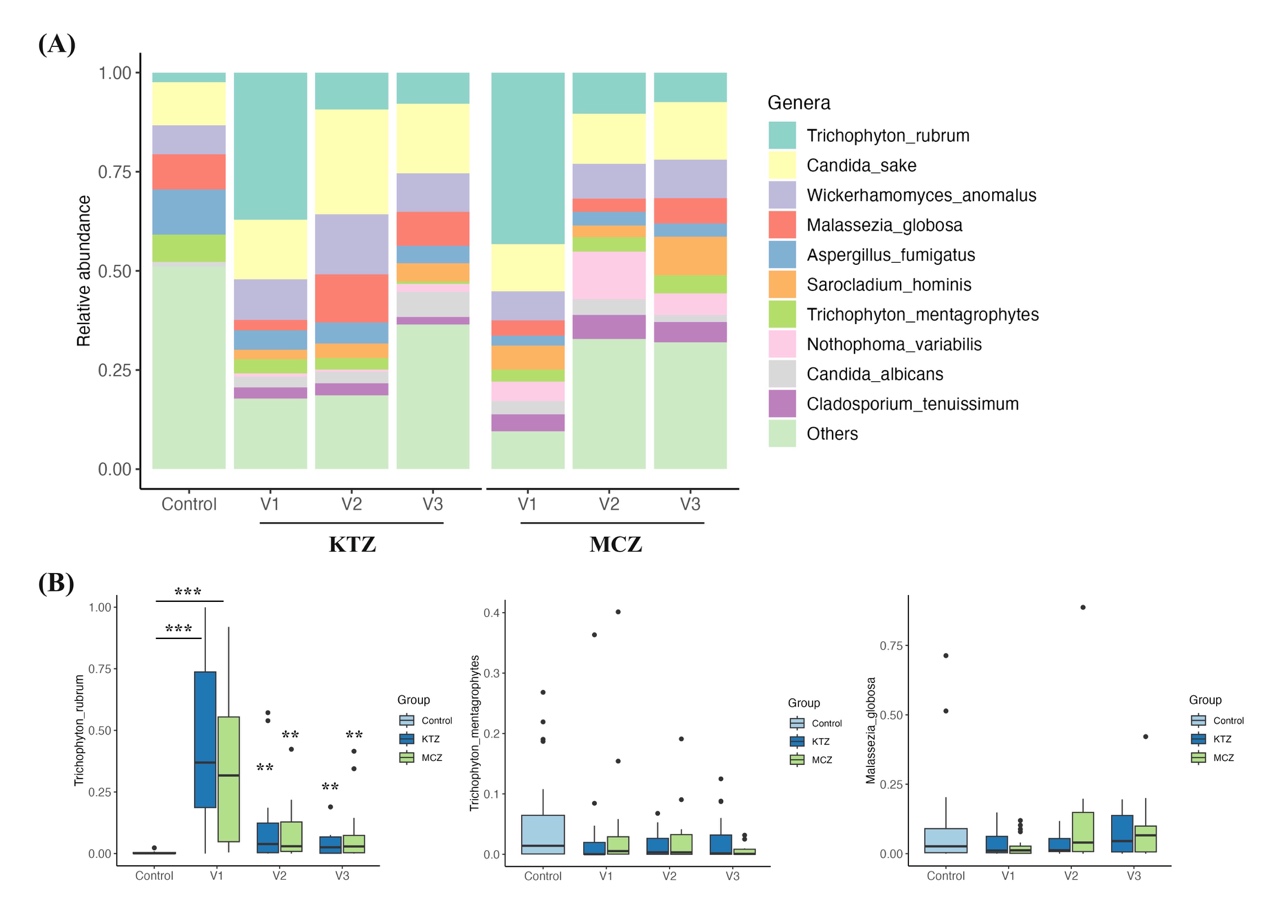


Supplementary figure 3. (A) Skin mycobiome structures of patients with tinea pedis and controls at the species level across different time points. (B) The relative abundances of *Trichophyton rubrum*, *Trichophyton mentagrophytes* and *Malassezia globosa* in tinea pedis patients and controls at different time points.
